# Acetate-based syntrophy enhances methane production potential of ruminant feces

**DOI:** 10.3389/fmicb.2025.1706620

**Published:** 2025-11-10

**Authors:** Jian Liu, Yujie Sha, Run Dang, Lifeng Zhou, Meng Zhou, Yang Tan, Jinming Wang, Ge Ran, Wei Xie, Dong Xia, Luotong Wang, Xingtang Zhao, Bok-Min Goi, Jiafeng Yu, Leilei Xiao

**Affiliations:** 1 Shandong Engineering Research Center of Green and Efficient Breeding and Food Deep Processing of Featured Livestock and Poultry, Institute of Biophysics, Dezhou University, Dezhou, China; 2 International Joint Laboratory of Agricultural Food Science and Technology of Universities of Shandong, Dezhou University, Dezhou, China; 3 CAS Key Laboratory of Coastal Environmental Processes and Ecological Remediation, Yantai Institute of Coastal Zone Research, Chinese Academy of Sciences, Yantai, China; 4 State Key Laboratory of Black Soils Conservation and Utilization, Northeast Institute of Geography and Agroecology, Chinese Academy of Sciences, Harbin, China; 5 School of Food and Biological Engineering, Yantai University of Science and Technology, Yantai, China; 6 Dezhou Animal Husbandry and Veterinary Career Development Center (Dezhou Animal Disease Prevention and Control Center), Dezhou, China; 7 Lee Kong Chian Faculty of Engineering & Science, Universiti Tunku Abdul Rahman (UTAR), Kajang, Selangor, Malaysia

**Keywords:** methanogenesis, feces, isotope tracing, metagenome, acetoclastic pathway

## Abstract

Livestock feces contribute to approximately 32% of global methane emissions. Although ruminants are generally believed to have a higher methane production potential than non-ruminants, the dominant pathways and key regulatory processes underlying methane generation in ruminants remain poorly understood, impeding effective manure management and accurate livestock emission assessments. In this study, metagenomic and carbon isotope techniques were employed to investigate methane production potential and key pathways in sheep, pig, chicken, and duck feces. Methane production potential of ruminant sheep feces was significantly higher (approximately threefold) compared to that of non-ruminants. Isotopic analysis of methane sources revealed that sheep feces primarily produce methane through the acetoclastic pathway, whereas the other three likely rely on CO_2_ reduction. Metagenomic analysis of methanogenic pathways further indicated that the abundance of functional genes associated with acetoclastic methanogenesis is significantly higher in sheep feces compared to the other three. Moreover, the co-occurrence network analysis highlighted a tightly coordinated, cross-species partnership between fermentative acetogenic bacteria and methanogenic archaea in the sheep fecal microbiome. Together, our findings provide insights into some key methanogenic pathways, such as acetoclastic methanogenesis, contributing to high methane production from ruminant feces.

## 1 Introduction

As the second most impactful greenhouse gas after carbon dioxide, methane contributes approximately 16–25% of total anthropogenic greenhouse gas emissions worldwide, and has recently gained heightened scientific and policy focus due to its potent heat-trapping capacity—86 times more powerful than CO_2_ over a 20-year period (Lee et al., 2023; Rosentreter et al., 2021). Agriculture is the world’s largest anthropogenic methane emission source, accounting for approximately 41% of global anthropogenic methane emissions, with the livestock sector responsible for 32% of the global emissions (Wang et al., 2024). Methane emissions vary across different livestock species in animal husbandry (Food and Agriculture Organization of the United Nations (FAO), 2023). Generally, most global CH_4_ emissions from livestock are attributed to cattle husbandry (Hanafiah et al., 2021; Liu et al., 2018; Malik et al., 2023; Wolak et al., 2022), and robust methane production may be attributed to acetate metabolism (Liu et al., 2025). The population of other animals has generally surpassed that of cattle, for example, with the chicken population exceeding that of cattle by more than twentyfold (Food and Agriculture Organization of the United Nations (FAO), 2023). However, research on small-bodied livestock, such as sheep, pigs, ducks, and chickens, is limited. Understanding emissions from these livestock is essential to support stringent global mitigation goals (Reisinger and Clark, 2018).

Anaerobic digestion is a bioprocess that yields bio-methane from a variety of biomass substrates, primarily consisting of four processes: hydrolysis, acidogenesis, acetogenesis, and methanogenesis (Ma et al., 2021). Methane production during the anaerobic digestion process is closely related to the physiological properties of the ecological niche (Kaushal et al., 2022; Kunatsa and Xia, 2022; Zamri et al., 2021), especially the microbial community. The fermentation is mainly conducted by bacteria, such as members of the phyla of Bacteroidetes, Firmicutes, and Thermotogae. The methanogenic process is primarily conducted by archaea, such as Methanosarcina, Mehanobacterium, and Methanosaeta (Ma et al., 2021). Based on the types of substrates, methanogens can be divided into three different physiological categories (Bechtold et al., 2025): (1) hydrogenotrophic methanogens, which use H_2_/CO_2_ as a substrate for the formation of methane; (2) methylotrophic methanogens, which produce methane by using methylated substrate to reduce carbon dioxide; (3) acetoclastic methanogens, which use acetate as a substrate for methanogenesis. Therefore, understanding the microbial community and its contribution to methane production is crucial for mitigation and ecological significance (Liu et al., 2018).

This study investigated the methane production potential and pathways of different animal feces based on experimental results from stable isotope analysis and metagenome-assembled genomes (MAGs). It is hypothesized that the methane production potential varies among different animal feces due to differences in their microbial composition and methanogenic pathways.

## 2 Materials and methods

### 2.1 Sample collection and cultivation experiments

Fresh feces were collected from different farms in Weifang, with at least six replicates for each type of animal. The experimental procedure was as follows: 1 g of fresh feces and 5 g of water were added to serum bottles, which were then incubated at 30 °C in the dark for 22 days. The serum bottles were subjected to vacuum/charging cycles with high-purity nitrogen (three cycles) to establish an anaerobic environment. Triplicate serum bottles were sacrificed to measure CH_4_ and acetate concentrations. Stable isotope analysis was performed on samples collected on day 22.

### 2.2 Chemical analysis

CH_4_, CO_2_, and acetate concentrations were measured using gas chromatography (GC; Agilent Technologies, USA) equipped with a flame ionization detector and high-performance liquid chromatography (Agilent Technologies, USA) equipped with a refractive index detector, respectively. The specific methods were performed according to the studies reported by Xiao et al. (2019).

### 2.3 Carbon stable isotope analysis

The determination of carbon stable isotopes in samples was conducted as follows: a gas chromatograph combustion isotope ratio mass spectrometer system (Thermo Fisher MAT253, Germany) was used for the measurement. CH_4_ and CO_2_ were first separated by a Finnigan PreCon (Thermo Fisher MAT253, Germany). The obtained CH_4_ was prefilled with helium gas and loaded into a chemical trap capable of removing CO_2_ and H_2_O, followed by oxidation to CO_2_ and H_2_O in a combustion reactor at 960 °C. The obtained CO_2_ was purified by two consecutive liquid nitrogen cold traps filled with Ni wires and subsequently analyzed by an isotope ratio mass spectrometer system (Thermo Fisher MAT253, Germany). The *δ* notation was used to denote the abundance of ^13^C as shown in the following equation (Gehring et al., 2015; Xiao et al., 2019):


δ13C=[(1213C)sample(2212C)PDB−1]×1000

where PDB represents Pee Dee Belemnite. The determination of δ^13^C-CO_2_ was similar, where a water trap replaced the chemical trap. The *α*-value was calculated as expressed in the following equation (Gehring et al., 2015; Xiao et al., 2019):


$$\alpha =\frac{\delta^{13}CO_{2}+1000}{\delta^{13}CH_{4}+1000}$$

### 2.4 DNA extraction, pyrosequencing, and data processing

Samples were collected for metagenomic sequencing on day 22. Total DNA was extracted using E. Z. N. A.® Soil DNA Kit (Omega Bio-tek, Norcross, GA, United States). A paired-end library was constructed using NEXTflexTM Rapid DNA-Seq Kit (Bio Scientific, Austin, TX, United States). Adapters containing the full complement of sequencing primer hybridization sites were ligated to the blunt ends of DNA fragments. The library was pooled and sequenced on Illumina HiSeq Xten for sequencing (Illumina, Inc., San Diego, CA, United States) at OEBiotech Co., Ltd. (Qingdao, China). The raw data were deposited in the National Microbiology Data Center (Beijing, China) under the accession number: NMDC10018765.

### 2.5 Metagenomic data assembly

Metagenomic sequences were *de novo* assembled using a Linux server equipped with 88 CPU cores and 1 TB of RAM. Trimmomatic was employed to remove adapters and filter low-quality reads (Bolger et al., 2014). Contaminations were removed by aligning the reads to the Viridiplantae genome (taxid: 33090) and the human genome Hg38 using BBmap. MEGAHIT was used to co-assemble the final clean reads from each sample (Cao et al., 2025; Li et al., 2015). Contigs larger than 500 bp were further used for later analysis. The quality of the assembly was evaluated using QUality ASsessment Tool (QUAST) (Gurevich et al., 2013).

### 2.6 Genomic binning and annotation

Genomic binning and annotation were conducted as follows: the binning process was performed using MetaWRAP with default parameters (Uritskiy et al., 2018). The resulting MAGs were filtered using the bin_refinement module with completeness > 50% and contamination < 10%. Each MAG was normalized to copies per million reads. Taxonomic annotation of the MAGs was performed using Genome Taxonomy Database Toolkit (GTDB-Tk) (version 2.2.0, Chaumeil et al., 2020). The prediction and annotation of open reading frames (ORFs) in MAGs were performed using Prodigal (Hyatt et al., 2010) and EggNOG-Mapper with default parameters.

### 2.7 Phylogenetic analysis

The genomes were subjected to GTDB-Tk (Chaumeil et al., 2020) to align the single-copy genes. The concatenated alignments were used to construct the phylogenetic tree with FastTree (Price et al., 2009). The phylogenetic tree was visualized and annotated using the Interactive Tree of Life (iTOL) web platform.^1^

### 2.8 Composition analysis

The microbial composition was assessed at the read level using Kraken2 (Wood et al., 2019), with details consistent with our previous study (Liu et al., 2023). Principal coordinate analysis (PCoA) of Bray–Curtis distances was performed using the R package vegan, based on the relative abundance of species.

### 2.9 Co-occurrence network analysis

Co-occurrence networks of MAGs (with an average abundance > 1 copies per million reads) were constructed using the R packages Hmisc and igraph. Downstream analysis included only species exhibiting significant correlations. Spearman’s correlation coefficient was calculated, and species with adjusted *p*-values < 0.05 and *R*-values > 0.6 were retained for downstream analysis (Kajihara and Hynson, 2024).

### 2.10 Statistical analysis

Differences in methane yield, *α*-values, δ^13^C values, and acetate concentration among species were assessed using a one-way analysis of variance. Differences were considered statistically significant at *p*-value < 0.05. Differential abundance of taxa (or genes) across animals was identified using the limma package (version 3.46.0). Raw count data were log2-transformed after the addition of 0.0000001. A linear model was fitted using the lmFit function, incorporating a design matrix that accounted for the grouping factor. Empirical Bayes moderation was applied with the eBayes function (with trend = FALSE). Differences in microbial community structure between animal feces were assessed using permutational multivariate analysis of variance (PERMANOVA) implemented in the adonis2 function of the vegan package (version 2.6–4), based on a Bray–Curtis distance matrix with 999 permutations. Following a significant global test, pairwise comparisons were performed using the pairwise.adonis2 function (pairwiseAdonis package, version 0.4.1). The resulting *p*-values from all comparisons were adjusted for multiple testing using the Benjamini–Hochberg false discovery rate (FDR) method. Significantly differentially abundant features were defined as those with an FDR-adjusted *p*-value < 0.05.

## 3 Results

### 3.1 CH_4_ production and stable isotopic analysis of different animal feces

**Figure 1:**
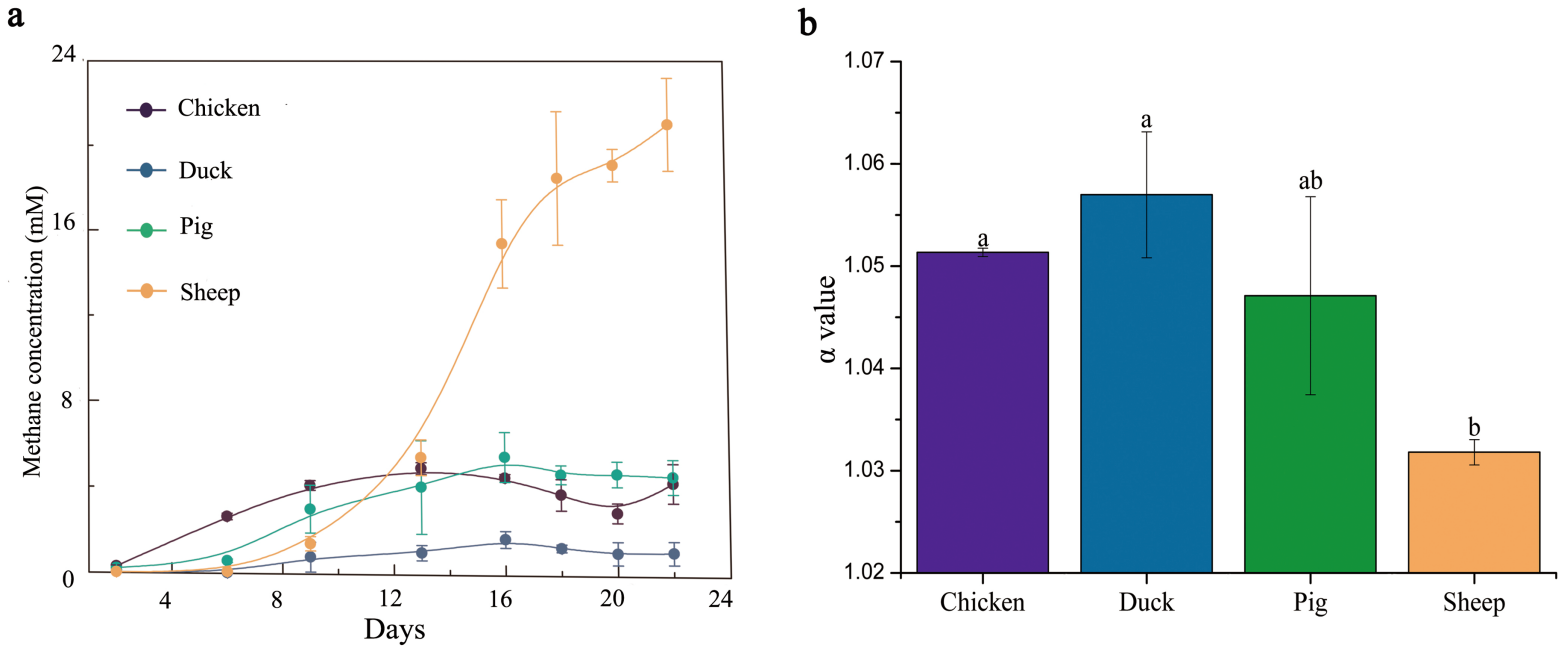
CH_4_ production and *α*-value of different animal feces at room temperature. CH_4_ production kinetics **(a)** was determined during 22 days. *α* value **(b)** was measured on the 22nd day. The error bar represented the standard error.

Feces from four different animal species used in this study exhibited distinct CH_4_ production potentials (Figure 1a). Under the same experimental conditions, sheep feces exhibited the highest CH_4_ production potential, with a maximum CH_4_ yield of 21 mM. This was approximately 3.5-fold greater than that of pig and chicken feces (approximately 6 mM) and a substantial 21-fold increase compared to duck feces (1 mM).

To link CH_4_ production to its precursors, *α*-values and δ^13^C values of CH_4_ were analyzed at the end of the experiment (Figure 1b; Supplementary Figure S1a), and acetate concentrations were measured over 22 days (Supplementary Figure S1b). The α-values of sheep feces were below 1.030, around 1.047 in pig feces, and above 1.050 in chicken (1.051) and duck feces (1.058) at the end of the experiment (Figure 1b). The δ^13^C-CH_4_ values were approximately −40‰ in sheep feces, while in other species they were higher than −55‰ (Supplementary Figure S1a).

### 3.2 Microbiome composition

**Figure 2:**
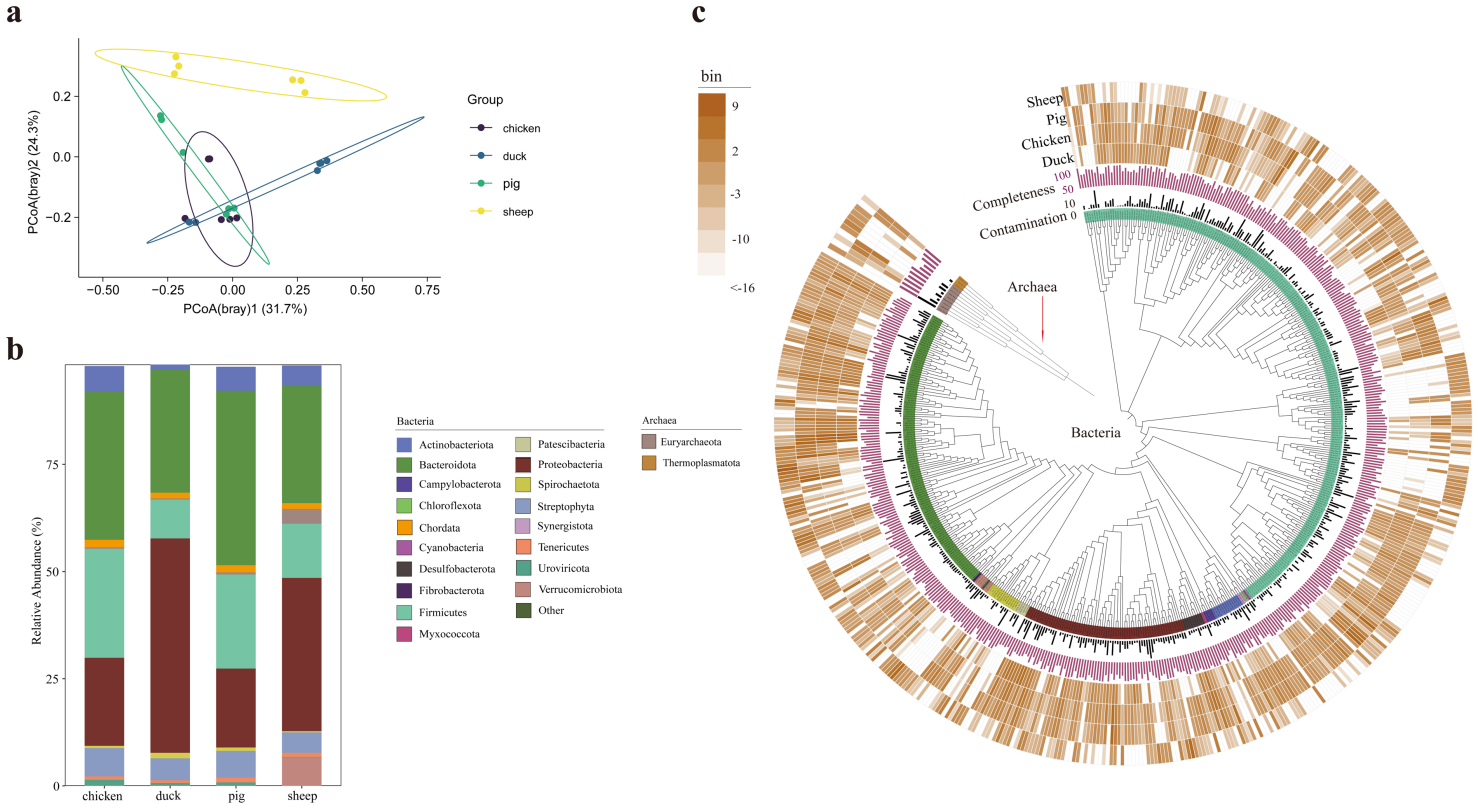
Microbial community composition and phylogenetic analysis of animal fecal samples. The principal coordination analysis of samples based on Bray–Curtis distances of reads classification (*n* = 6) **(a)**. Taxonomic composition showing the dominant phyla with relative abundance above 1% **(b)**. Maximum likelihood phylogenomic tree of the bacterial and archaeal MAGs **(c)**. The bar layers depict genome quality metrics [purple: completeness (%); dark gray: contamination (%)]. The outer four concentric heatmaps represent log_2_(bin abundance) (copies per million reads) profiles in sheep, pig, chicken, and duck feces, respectively. The color stripe in the inter-ring layer showed the phylum levels.

To further explore the underlying mechanisms of differential methane production in different animal feces, the microbial community was analyzed based on high-quality reads. Microbial alpha diversity indexes had no significant differences among the animals (Supplementary Figure S2). The principal coordinate analysis (PCoA) based on Bray–Curtis distances revealed that the samples from sheep were significantly distinct from other samples (pairwiseAdonis: all adjusted *p*-values = 0.0097, Figure 2a). At the phylum level, sheep feces exhibited the highest proportion of Euryarchaeota (3.46%, *p* < 0.05), followed by pig feces (0.50%), chicken feces (0.33%), and duck feces (0.27%) (Figure 2b; Supplementary Figure S3).

Metagenomic binning is the process of grouping metagenomic sequences based on their organism of origin (Nissen et al., 2021). In metagenomic studies, binning facilitates the reconstruction of known and unknown genomes, enabling in-depth exploration of microbiome variation at the species level (Liu et al., 2023; Nissen et al., 2021). A total of 511 unique bacterial and archaeal metagenome-assembled genomes (MAGs) were recovered through the co-assembly step, with completeness > 50% and contamination < 10%. Among these, 500 MAGs were classified as bacteria, with the predominant phyla being Firmicutes (249), Bacteroidetes (124), Proteobacteria (69), Spirochaetota (14), and Actinobacteria (13) (Figure 2c). The remaining 11 MAGs were classified as archaea including Euryarchaeota (8) and Thermoplasmatota (3) (Figure 2c). Notably, four MAGs were only existed in sheep fecal samples (sheep.bin.3, sheep.bin.10, sheep.bin.84, and sheep.bin.185).

### 3.3 Potential methanogenesis pathways in different animal feces

**Figure 3:**
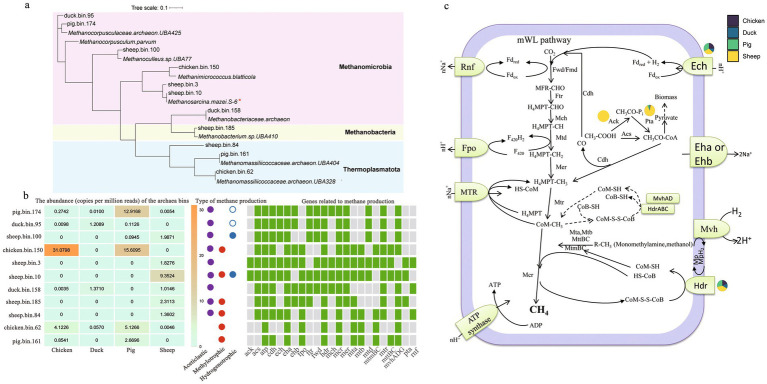
Potential functional microbes and metabolism for CH_4_ production. Maximum likelihood phylogenomic tree showing the closest classification of potential methanogenic MAGs **(a)**. Evaluation of the genes related to methane production and the abundance of methanogens (*n* = 6) **(b)**. Only the MAGs with a relative abundance above 1% of the total archaea were shown. Green and grey boxes showed the presence and absence of the genes in a genome, respectively. Colored circles: aceticlastic methanogenesis (purple); methylotrophic methanogenesis (red); hydrogenotrophic methanogenesis (blue). Open circles indicated predicted H_2_-dependent methylotrophic methanogenesis. Complete methanogenic pathways and closely related genes **(c)**. Pie charts denoted the relative abundances of differentially abundant methanogenic genes across animals. Gene abbreviations are detailed in the Supplementary materials.

A phylogenetic analysis of the 11 archaeal MAGs was performed (Figure 3a). Species belonging to the class Methanomicrobia were the most abundant archaea across all fecal samples. Archaeal bins were most abundant in pig feces [36.53 copies per million reads (CPM)], followed by chicken feces (36.34 CPM), sheep feces (17.86 CPM), and duck feces (2.65 CPM) (Figure 3b).

**Figure 4:**
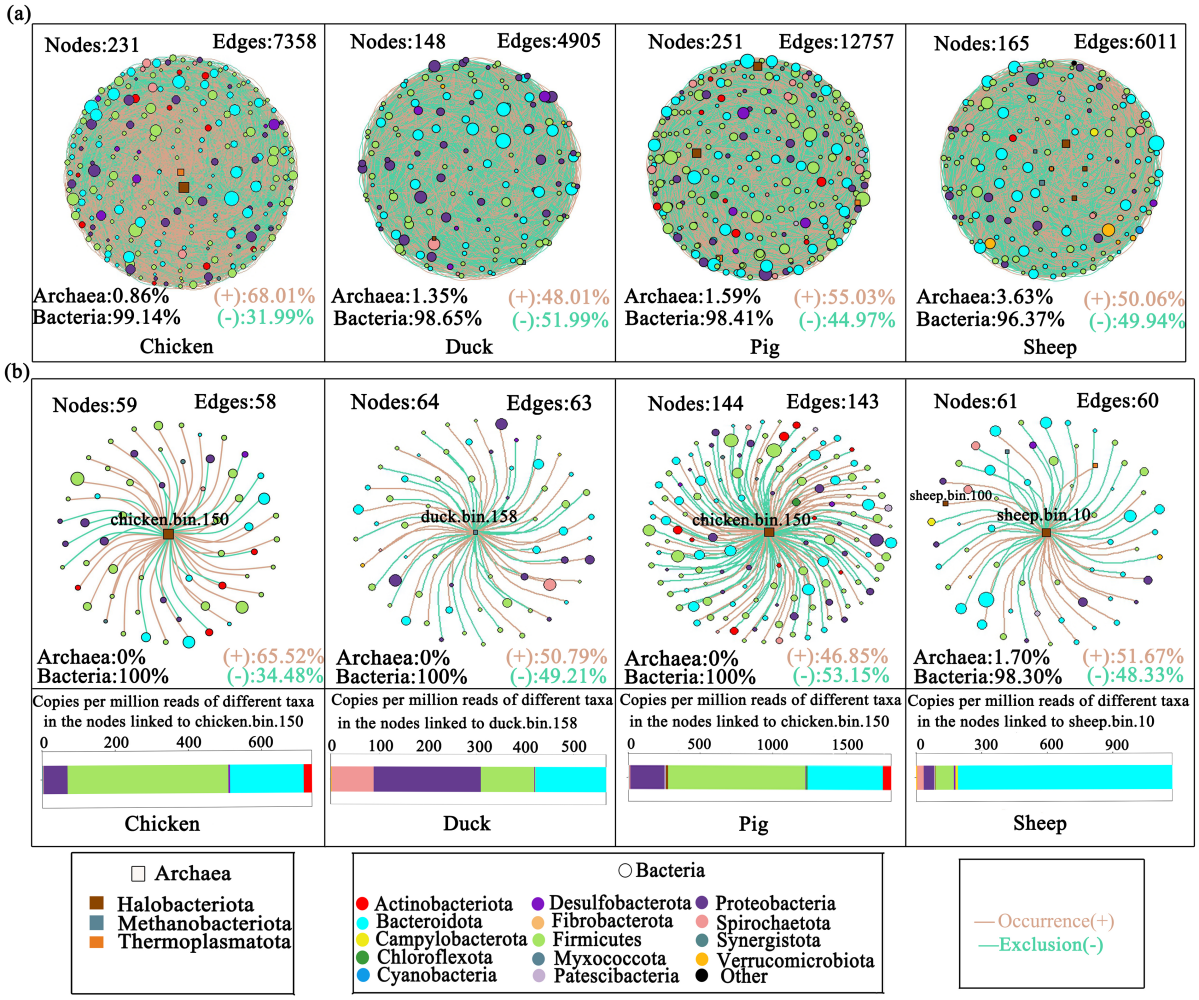
Co-occurrence networks of MAGs. Networks of bacterial and archaeal MAGs in each animal **(a)**. Sub-networks of dominant MAGs in each animal **(b)**. The size of nodes in each network is proportional to the relative abundance of its phylum. Different node colors denote different phyla. Squares denote archaea, while circles denote bacteria. Bar plots showed the copies per million reads of different taxa in the nodes linked to the most abundant MAG in each animal feces. Green lines denote exclusion (−). Orange lines denote co-occurrence (+).

Sheep.bin.10, identified as *Methanosarcina mazei*, was the most dominant methanogenic archaeon (9.35 CPM) and only existed in sheep fecal samples (Figures 3a,b). It contained all genes involved in acetoclastic methanogenesis, indicating that this MAG could utilize acetic acid to produce CH_4_. The second most abundant methanogen (2.31 CPM), sheep.bin.185, also utilized acetic acid to produce CH_4_ (Figures 3b,c). Sheep.bin.100, identified as *Methanoculleus* sp., can also utilize acetic acid (Figure 3), and exhibited a direct co-occurrence relationship with sheep.bin.10 (Figure 4b). Additionally, sheep.bin.3, sheep.bin.84 and sheep.bin.185, theses MAGs only existed in feces of sheep and possessed the potential to utilize acetic acid to produce CH_4_ (Figures 3b,c). This may explain the low acetate content observed in sheep fecal samples (Supplementary Figure S1b). Therefore, combined with isotopic analysis, acetoclastic pathways are the primary contributors to methane production in sheep fecal samples.

Chicken.bin.150, identified as *Methanimicrococcus blatticola*, was the dominant archaeal MAG in chicken (31.08 CPM) and pig fecal samples (15.61 CPM). Despite the presence of genes involved in the acetoclastic methanogenesis pathway in chicken.bin.150, isotopic analysis and acetate utilization results indicated that this MAG may exhibit little to no capacity for acetate utilization (Figure 1b; Supplementary Figure S1b). Additionally, Chicken.bin.150 has lost nearly all genes coding for the H_4_MPT methyl branch of the Wood-Ljungdahl pathway, except for *mer* (Figures 3b,c). A similar phenomenon was also observed in pig.bin.174, the second most abundant MAGs in pig fecal samples. Duck.bin.158, identified as *Methanobacteriaceae archaeon*, was the most dominant methanogenic archaeon in the duck fecal samples (1.37 CPM). However, the abundance of *Methanobacteriaceae* was rather low, which may explain the lower CH_4_ production potential of these samples.

Based on contigs-based profiling of methanogenic genes, significant disparities in relative abundance (*p* < 0.05) were observed across animal fecal samples for only four genes: *ack*, *pta*, *ech*, and *hdr* (Figure 3c). The *ack* gene was uniquely present in sheep feces, with *pta* abundance exceeding that in other species by at least 10-fold (Supplementary Table S1), indicating a distinct acetoclastic methanogenic pathway in sheep-derived archaea. Moreover, sheep feces exhibited markedly higher *hdr* gene abundance (*p* < 0.05), underscoring a potentially pivotal role of electron transport in acetotrophic methanogenesis (Liu et al., 2023). Although *ech* gene abundance in sheep was significantly elevated compared to ducks, it did not differ significantly from other species.

### 3.4 Co-occurrence networks for bacterial and archaeal genomes

Co-occurrence networks of bacterial and archaeal genomes from four different animal species were constructed (Figure 4a). A total of 231, 148, 251, and 165 nodes were retrieved in the chicken, duck, pig, and sheep fecal samples, respectively, with archaeal proportions increasing from 0.86 to 3.63%. In chicken, pig, and sheep fecal samples, more than half of the edges represented co-occurrence, indicating that microorganisms predominantly coexisted and cooperated rather than competing (Miao et al., 2023).

Sub-networks between bacterial and the most abundant methanogenic MAGs in each animal were further analyzed (Figure 4b). In the sub-network of pig fecal samples, more than half of its edges represented in co-exclusion, indicating stronger competitive interactions (Zhang et al., 2022). Although similar numbers of nodes and edges were identified in sub-networks of chicken, duck and sheep feces, archaea were only found in linked bins of sheep feces, with a proportion of up to 1.7% (Figure 4b). Strikingly, sub-network analysis revealed 14 nodes with significantly greater abundance in the sheep feces compared to others (*p* < 0.05), including 9 Clostridia-affiliated nodes that displayed robust co-occurrence relationships with sheep.bin.10 (Spearman’s *r*-value > 0.6) (Supplementary Table S3).

## 4 Discussion

Methane exerts a substantial influence on Earth’s climate due to its potent greenhouse effect. Identifying its major sources is essential for developing effective mitigation policies. In the agricultural sector, livestock represent a major source of methane emissions, primarily through enteric fermentation (Wang et al., 2024). Methane emissions vary significantly across animal species. Notwithstanding that the majority of global livestock-related methane emissions are attributable to cattle (Liu et al., 2025), research focusing on small-bodied species—such as sheep, pigs, ducks, and chickens—remains limited. The substantial population sizes of these species, which often surpass that of cattle by several folds, coupled with their non-negligible methane emission potential, warrant greater scientific attention. Elucidating these variations is crucial to support stringent global mitigation goals. This study investigated methane production potential and key pathways in sheep, pig, chicken, and duck feces by metagenomic and carbon isotope techniques. The results generally supported our core hypothesis that the methane emission potential varies among different animal feces due to differences in their microbial composition and methanogenic pathways.

In the present study, sheep fecal samples exhibited the highest methane production potential, exhibiting a > 3-fold increase compared to other species (*p* < 0.05). The δ^13^C-CH4 and *α*-values are often served as diagnostic indicators for distinguishing methanogenic pathways. The classic range for the δ^13^C-CH4 of the aceticlastic methanogenesis pathway is from −27‰ to −60‰, with a classic α-value range of 1.00–1.032 (Liu et al., 2023; Vinson et al., 2017; Whiticar, 1999). The natural abundance of carbon isotope fractionation in sheep feces exhibited typical δ^13^C-CH_4_ and α-values consistent with acetoclastic methanogenesis, implying that microorganisms in sheep feces preferentially produced methane through acetate disproportionation. This speculation was preliminarily supported by the results in Supplementary Figure S1b, where acetate could be utilized by microorganisms in sheep feces but not in chicken, duck, and pig feces.

Reported α-values exceeding 1.058 have been linked to more negative δ^13^C-CH4 signatures, interpreted as indicative of predominantly hydrogenotrophic methanogenesis (Vinson et al., 2017). This is consistent with findings in duck feces, where both α-values and the profile of methane-producing genes suggest a preference for hydrogenotrophic methanogenesis (Figure 3b). Although the classical α-value range for hydrogenotrophic methanogenesis was estimated as 1.049–1.095 (Whiticar, 1999), overlaps with methylotrophic methanogenesis have been documented (Penger et al., 2012). Accordingly, despite the analysis of methanogenic archaeal genes in chicken and pig feces (Figures 3b), the dominant methanogenic pathways in these hosts remain unresolved. Acetoclastic methanogenesis can be conclusively ruled out as a major pathway in sheep feces, but further validation of these observations is necessary, and the mechanisms underlying the differences in methanogenic pathways across hosts warrant deeper investigation.

Gut microbiota plays a crucial role in methane production through intestinal fermentation (Chen et al., 2025). Microbial diversity in feces is considered as a potential good indicator when exploring the intestinal ecosystems of hosts (Sha et al., 2024). In the present study, microbial alpha diversity indexes, which reflect the complexity and evenness of the microbial community (Hultman et al., 2015), showed no significant differences among the animals (Supplementary Figure S2), indicating no statistically significant variation in microbial alpha diversity across the four animal feces. Firmicutes and Bacteroidetes were the top two phyla in the feces of all four animal species. They serve as pivotal primary fermenters, hydrolyzing recalcitrant polymers to generate acetate, H_2_, and CO_2_. These products are directly utilized by methanogenic archaea, which act as the ultimate methane producers. This intricate microbial synergy is fundamental to efficient methane biogenesis (Bechtold et al., 2025). Notably, sheep feces exhibited the highest proportion of Euryarchaeota (3.46%; *p* < 0.05), showing a marked increase of over 6-fold compared to other species. The favorable CH_4_ production performance may be attributed to the higher abundance of methanogens within the Euryarchaeota phylum (Liu et al., 2023).

Archaeal MAGs were recovered through the co-assembly step, among these, phylum Euryarchaeota were indeed the most abundant archaea across all fecal samples (Figure 3a). Interestingly, the highest relative abundance of Euryarchaeota was observed not in sheep feces, but in chicken feces. Archaea are responsible for over half of global annual methane emissions, and their methane-metabolizing genes play a critical role in this process (Evans et al., 2019). Analysis of methanogenic pathways across different animal feces revealed that MAGs encoding acetoclastic methanogenesis were predominantly enriched in sheep feces. This finding aligns with our previous research, which also identified acetoclastic methanogenesis as the primary methane-producing pathway in cattle (Liu et al., 2025). In contrast, other ruminants such as yaks and camels predominantly rely on methylotrophic and hydrogenotrophic methanogenesis (Mi et al., 2024). In sheep feces, representative MAGs including Sheep.bin.3, Sheep.bin.10, Sheep.bin.84, and Sheep.bin.185 were identified, among which Sheep.bin.10 was taxonomically classified as *M. mazei*, a known primary aceticlastic methanogen (Chang et al., 2024). The loss of both ack and pta genes in dominant archaeal MAGs (e.g., chicken.bin.150) from chicken and pig feces definitively precludes acetoclastic methanogenesis via the classic acetyl-CoA cleavage pathway (Mi et al., 2024), despite the presence of other related genes. Instead, these archaea may primarily rely on molecular hydrogen and methylated single-carbon compounds (e.g., methanol, methylated amines) for energy (Feldewert et al., 2020; Sprenger et al., 2007). This methanogenesis pathway may be related to its colonization in environments where both methyl compounds and hydrogen are abundant (Thomas et al., 2021). However, genes encoding hydrogenase were absent in chicken.bin.150 and its associated bins (Supplementary Figure S4; Supplementary Table S2), indicating H_2_ production rates in the environment may limit the methane production rate of chicken.bin.150 in chicken and pig fecal samples.

Ecological relationships (both co-occurrence and co-exclusion) among microbial inhabitants are important contributors to the variation in host microbiota, as they facilitate the exchange or competition for nutrients or signaling molecules (Ma et al., 2020). Analysis of sub-networks between bacterial and the most abundant methanogenic MAGs in each animal revealed that archaeal linkages were exclusively detected among co-occurring bins from sheep fecal samples. Specifically, Sheep.bin.100 demonstrated a co-occurrence relationship with Sheep.bin.10 (*M. mazei*), suggesting that their potential mutualistic interaction may be a key factor contributing to high methane production in sheep feces. Furthermore, phylogenetically associated bacteria play an essential role in anaerobic systems by hydrolyzing complex organic compounds into monomers. These monomers are subsequently fermented by specialist bacteria to produce energy-rich substrates (e.g., hydrogen, acetate, and formate) that support archaeal methanogenesis. For instance, Clostridium—a genus widely recognized for its role in lignocellulose degradation and acetate production (Amin et al., 2021)—was identified as a key participant in this trophic network. In particular, sub-network analysis revealed that nodes classified under Clostridia exhibited strong co-occurrence patterns (Spearman’s *r* > 0.6) with the archaeal MAG sheep.bin.10 (Supplementary Table S3). It is well established that microbial metabolic complementarity underpins the efficiency of anaerobic digestion, and the structural dynamics of microbial co-networks are critical determinants of process stability and methanogenic performance (Kajihara and Hynson, 2024). Accordingly, our findings indicate that the elevated methane production observed in sheep fecal samples arises from a tightly coordinated syntrophic interaction between fermentative Clostridia, which supply acetate, and methanogenic archaea, which consume it. This cross-kingdom partnership provides a mechanistic explanation for the high methane emissions typically associated with ruminant systems. To our knowledge, this study provides novel evidence, through an integrated metagenomic and carbon isotope approach, linking acetoclastic methanogenesis to high methane production potential in sheep feces—a relationship that has not been previously explored in such detail.

## 5 Conclusion

Sheep fecal samples demonstrated the highest methane production potential (MPP), exhibiting a > 3-fold increase compared to other species (*p* < 0.05). Isotopic analysis confirmed acetate as the predominant methanogenic precursor in sheep feces (δ^13^ C-CH4 ~ -40‰), with metagenomic evidence revealing acetoclastic methanogenesis as the dominant pathway (the abundance of *Methanosarcina* spp. ~9.35 CPM). Microbial co-occurrence sub-network analysis identified robust syntrophy between *Clostridia* (sheep.bin.115/bin.126/bin.129, etc.) and acetoclastic archaea (sheep.bin.10), functionally coupling acetate oxidation with methanogenesis. These results establish that acetoclastic methanogenesis may be the key driver of hypermethanogenesis in ruminant systems.

## Data Availability

The datasets presented in this study can be found in online repositories. The names of the repository/repositories and accession number(s) can be found at: https://nmdc.cn/, NMDC10018765.

## Footnotes

^1^ https://itol.embl.de/upload.cgi
