# Supplementary figures and images for: Acetate-based syntrophy enhances methane production potential of ruminant feces
Source: Front Microbiol. 2025 Nov 10;16:1706620. doi: 10.3389/fmicb.2025.1706620 (PMC12643385; doi:10.3389/fmicb.2025.1706620)

a

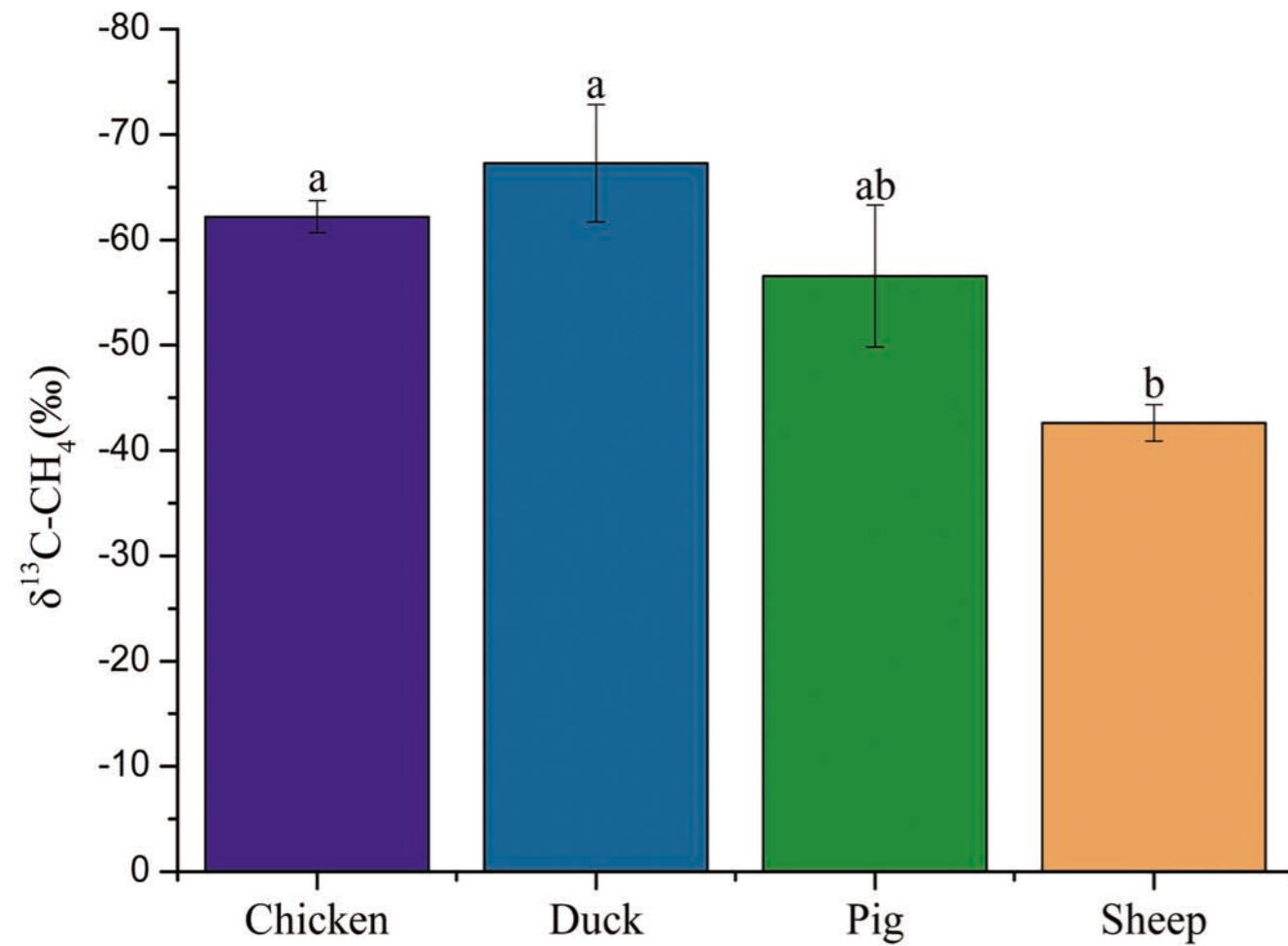

b

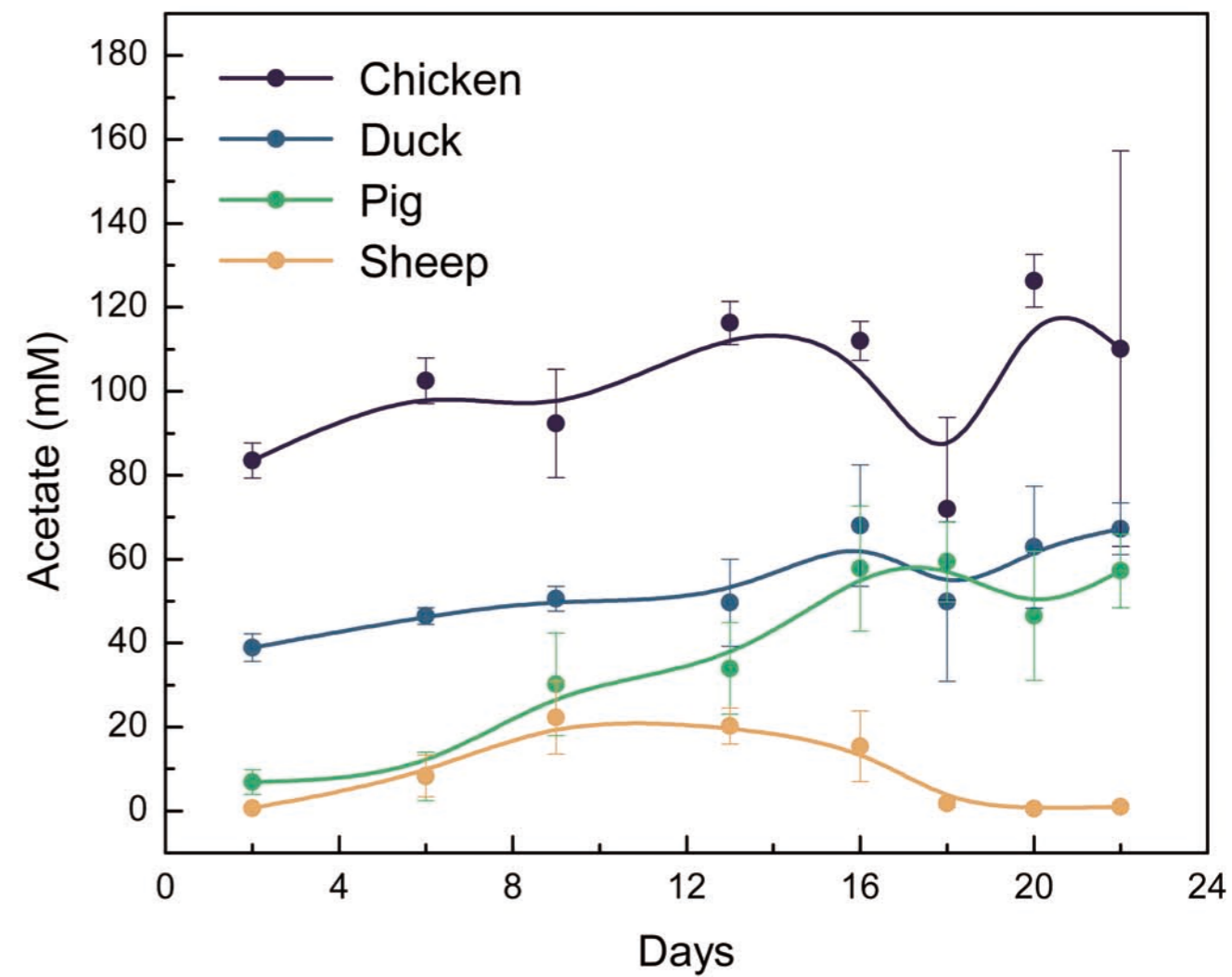

Supplement: Supplementary file 1 [file Image_1.pdf]

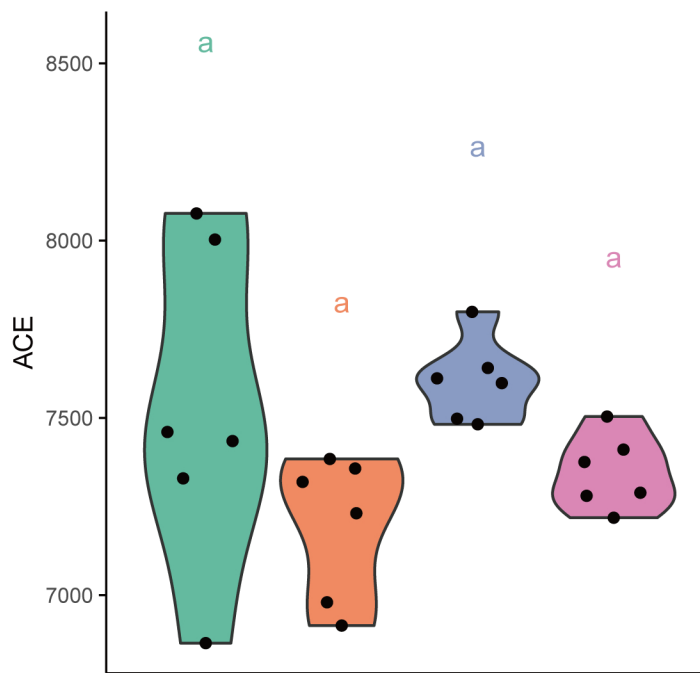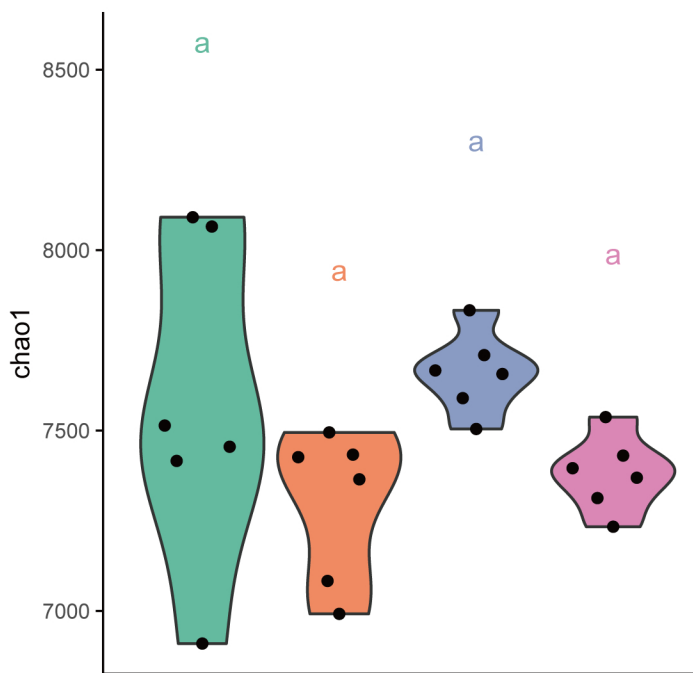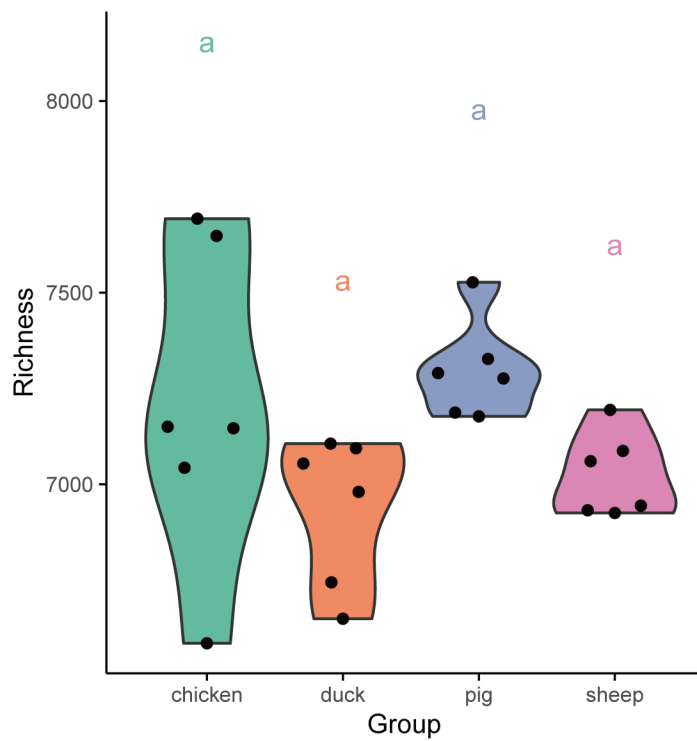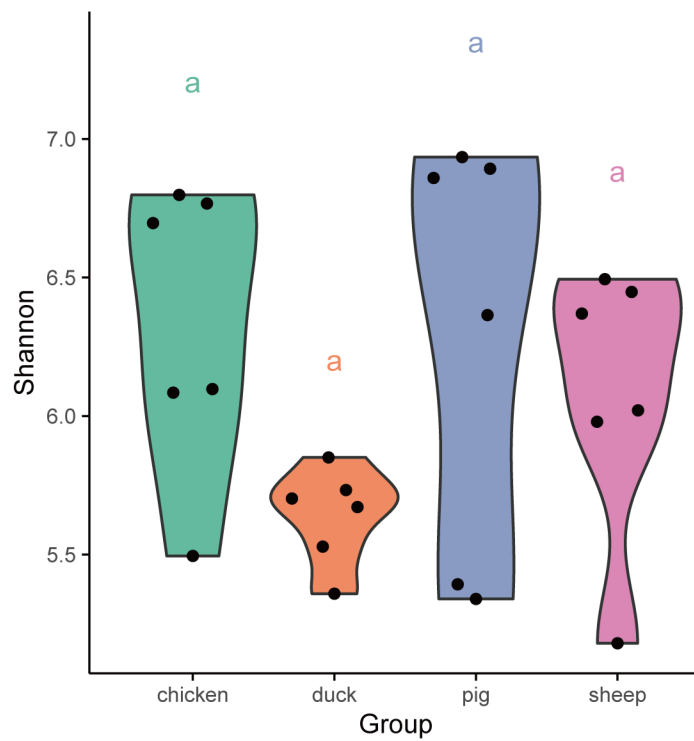

Supplement: Supplementary file 2 [file Image_2.pdf]

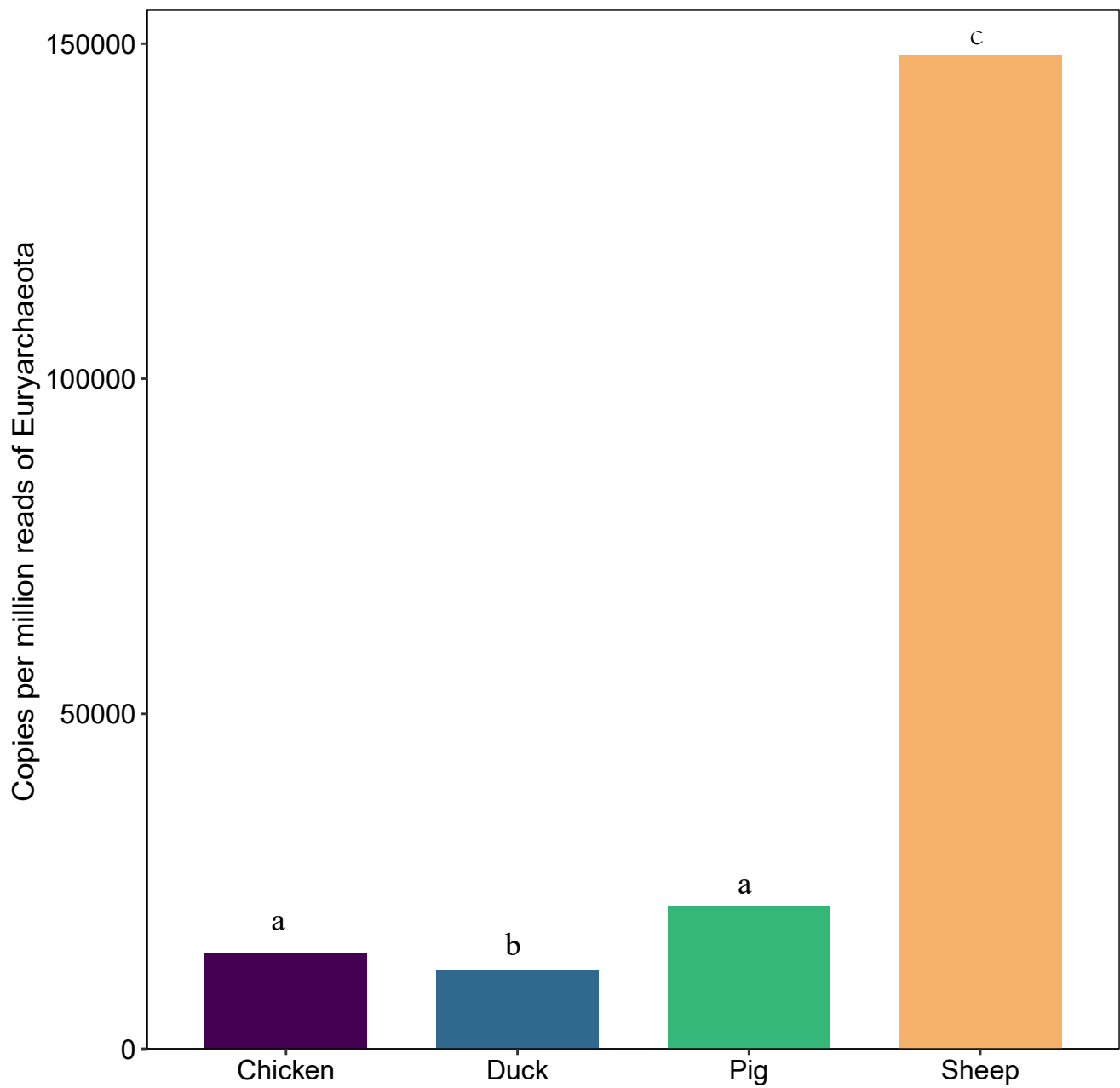

Supplement: Supplementary file 3 [file Image_3.pdf]

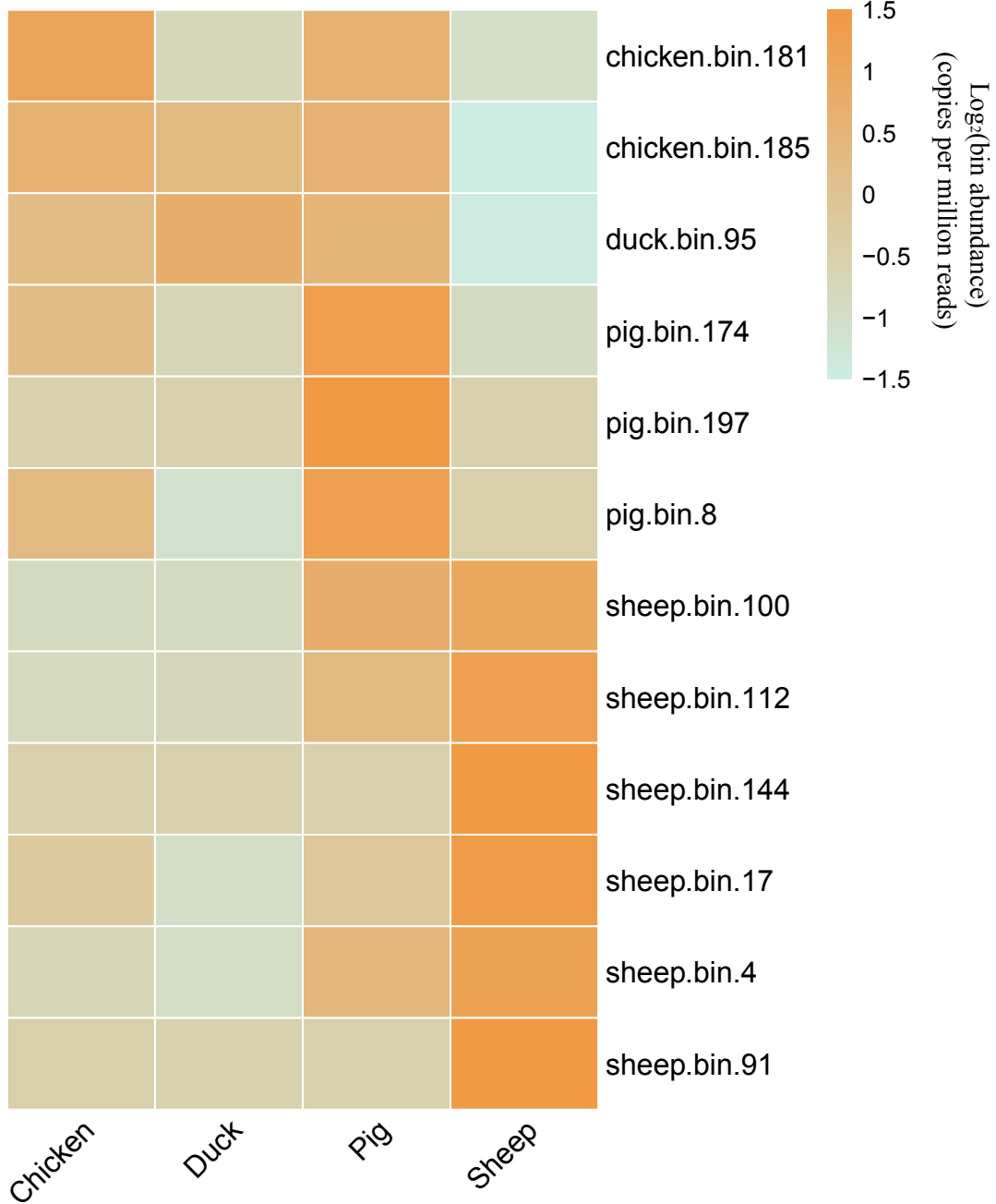

Supplement: Supplementary file 4 [file Image_4.pdf]
