# Supplementary material for: Acetate-based syntrophy enhances methane production potential of ruminant feces
Source: Front Microbiol. 2025 Nov 10;16:1706620. doi: 10.3389/fmicb.2025.1706620 (PMC12643385; doi:10.3389/fmicb.2025.1706620)
Supplement: Supplementary file 8 [file Data_Sheet_1.pdf]

The summary of metagenome sequencing data:

In this study, sequencing generated 2,011.76 million reads, amounting to a total of 301.76 Gb. The resulting data demonstrated high quality (Q20: 98.08% ±0.12%; Q30: 94.11% ±0.33%) and an average GC content of 42.63% ± 3.83%.

Fig.3 The abbreviation of genes. Ack, acetate kinase; Acs, acetyl-CoA synthase; atp: ATP synthase; Cdh, carbon monoxide dehydrogenase-acetyl-CoA synthase complex; Ech, energy-conserving hydrogenase; Eha and Ehb, energy-converting hydrogenase A and hydrogenase B; Fpo, F420-H_2-dehydrogenase; Ftr, formylmeth-anofuran-H4MPT formyltransferase; Fwd, formyl-methanofuran dehydrogenase; H4MPT, tetrahydromethanopterin; Hdr, heterodisulfide reductase; Mch, methenyl-H4MPT cyclohydrolase; Mcr, methyl-coenzyme M reductase; Mer, F420-dependent methylene-H4MPT reductase; Mta, including MtaA to MtaC, MtaA: [methyl-Co(III) methanol/glycine betaine-specific corrinoid protein]:coenzyme M methyltransferase, MtaB: methanol---5-hydroxybenzimidazolylcobamide Co-methyltransferase, MtaC: methanol corrinoid protein; Mtb, including MtbA to MtbC, MtbA: [methyl-Co(III) methylamine-specific corrinoid protein]:coenzyme M methyltransferase, MtbB: dimethylamine---corrinoid protein Co-methyltransferase; MtbC: dimethylamine corrinoid protein; Mtd, F420-dependent methylene H4MPT dehydrogenase; MtmBC, methylamine---corrinoid protein Co-methyltransferase and monomethylamine corrinoid protein; Mtr, including MtrA to MtrH, tetrahydromethanopterin S-methyltransferase subunit A-H; MttBC, trimethylamine---corrinoid protein and its Co-methyltransferase; MvhADG, F420-non-reducing hydrogenase subunits; Pta, phosphate acetyltransferase; Rnf, Na+-translocating ferredoxin-NAD oxidoreductase.

Fig.S1 CH_4 production and acetate utilization scenario of different animal feces at room temperature. $\delta^{13}C$-CH_4 (a) was measured at 22nd day. Acetate utilization kinetics (b) was determined during 22 days.

Fig.S2 The α diversity index of each group obtained from reads classification (n

6).

Fig.S3 The copies per million reads of Euryarchaeota in each group.

Fig.S4 The heatmap of MAGs containing potential genes coding for hydrogenase in each group.
